# Biceps Femoris Long Head Muscle Fascicles Actively Lengthen During the Nordic Hamstring Exercise

**DOI:** 10.3389/fspor.2021.669813

**Published:** 2021-06-09

**Authors:** Brent J. Raiteri, Ronja Beller, Daniel Hahn

**Affiliations:** ^1^Human Movement Science, Faculty of Sport Science, Ruhr University Bochum, Bochum, Germany; ^2^Department of Pediatric Hematology/Oncology, Center for Child and Adolescent Medicine, Essen University Hospital, Essen, Germany; ^3^School of Human Movement and Nutrition Sciences, The University of Queensland, Brisbane, QLD, Australia

**Keywords:** active stretch, biomechanics, eccentric, hamstrings, muscle lengthening, fascicle behavior, tendon compliance, ultrasound

## Abstract

Current debate exists around whether a presumed eccentric exercise, the Nordic hamstring exercise (NHE), actually causes active hamstring muscle lengthening. This is because of the decoupling that can occur between the muscle fascicle and muscle-tendon unit (MTU) length changes in relatively compliant human lower-limb MTUs, which results in MTU lengthening not necessarily causing muscle fascicle lengthening. This missing knowledge complicates the interpretation of why the NHE is effective at reducing running-related hamstring muscle injury risk in athletes previously unfamiliar with performing this exercise. The purpose of the study was therefore to investigate if the most-commonly injured hamstring muscle, the biceps femoris long head (BF), exhibits active muscle lengthening (i.e. an eccentric muscle action) during the NHE up until peak force in Nordic novices. External reaction force at the ankle, knee flexion angle, and BF and semitendinosus muscle activities were recorded from the left leg of 14 participants during the NHE. Simultaneously, BF muscle architecture was imaged using B-mode ultrasound imaging, and muscle architecture changes were tracked using two different tracking algorithms. From ~85 to 100% of peak NHE force, both tracking algorithms detected that BF muscle fascicles (*n* = 10) significantly lengthened (*p* < 0.01) and had a mean positive lengthening velocity (*p* ≤ 0.02), while knee extension velocity remained positive (17°·s^−1^) over knee flexion angles from 53 to 37° and a duration of 1.6 s. Despite some individual cases of brief isometric fascicle behavior and brief fascicle shortening during BF MTU lengthening, the predominant muscle action was eccentric under a relatively high muscle activity level (59% of maximum). Eccentric hamstring muscle action therefore does occur during the NHE in relatively strong (429 N) Nordic novices, which might contribute to the increase in resting BF muscle fascicle length and reduction in running-related injury risk, which have previously been reported following NHE training. Whether an eccentric BF muscle action occurs in individuals accustomed to the NHE remains to be tested.

## Introduction

Despite extensive research efforts, hamstring injuries remain a large burden for high-speed running athletes (Lysholm and Wiklander, 1987; Opar et al., 2014; Ekstrand et al., 2016). Hamstring injuries often involve the biceps femoris long head (BF) (Verrall et al., 2003; Woods et al., 2004; Askling et al., 2007), which may be at an increased risk of injury because of simulated data showing greater strain of its active muscle fibers during the late-swing phase of high-speed running relative to the other hamstring muscles (Chumanov et al., 2011). Consequently, considerable attention has been given to BF muscle injury prevention (Arnason et al., 2008; Petersen et al., 2011; Seagrave et al., 2014; van der Horst et al., 2015; Timmins et al., 2016).

One exercise that has received particular attention because of its reported effectiveness in reducing BF muscle injury risk in high-speed running athletes is the Nordic hamstring exercise (NHE) (Al Attar et al., 2017; van Dyk et al., 2019). The NHE is assumed to cause eccentric hamstring muscle action and thus be an eccentric exercise because there is a rightward shift in the optimum angle for torque generation to longer hamstring lengths immediately after and up to 10 days after an initial NHE bout. The NHE also induces a repeated-bout effect (Brockett et al., 2001), and training the exercise for 5–10 weeks increases muscle fascicle lengths of the BF (Bourne et al., 2017; Presland et al., 2018). However, despite this indirect evidence, there is no direct experimental support that the NHE causes eccentric hamstring muscle action, and assuming muscle fascicle length changes from muscle-tendon unit (MTU) length changes is complicated because of the decoupling that can occur due to MTU compliance (Griffiths, 1991). For example, despite MTU lengthening in the mid-stance phase of overground walking, relatively isometric soleus (SOL) muscle fascicle behavior has been observed (Cronin et al., 2013) because the SOL MTU is highly compliant (Zajac, 1989). Consequently, obtaining muscle fascicle length changes from the hamstrings (especially the BF MTU, which is also relatively compliant) (Zajac, 1989) during the NHE is needed to help justify the interpretation of why this exercise is effective in reducing running-related hamstring muscle injury risk.

To determine whether eccentric hamstring muscle action occurs during the NHE in Nordic novices, we imaged BF muscle fascicle lengths during the NHE using two-dimensional B-mode ultrasound imaging in combination with external reaction force, knee angle, and surface electromyography measurements from the hamstrings. BF muscle fascicle length changes were determined using two ultrasound tracking algorithms. Based on previous indirect evidence that eccentric hamstring muscle actions occur during the NHE, we hypothesized (1) that BF muscle fascicles would lengthen under moderate-to-high muscle activity levels during BF MTU lengthening in the NHE and (2) that the mean BF fascicle velocity would therefore be positive during this phase.

## Materials and Methods

### Participants

A sample size of 13 participants was estimated *a priori* to have 90% power to detect a minimum effect size (Cohen's *d*_*z*_) of 1 in a two-tailed, one-sample *t*-test between fascicle length change and a constant of zero. Consequently, 14 (allowing for one dropout/exclusion) healthy and athletic or recreationally-active male participants (age 26 ± 3 years, body mass 83 ± 9 kg, and height 1.84 ± 0.07 m) with no recent (<12 months) lower-limb injuries or surgeries were recruited and voluntarily participated in the study after providing written informed consent. The study was conducted in accordance with the Declaration of Helsinki and approved by the Faculty of Sport Science Ethics Committee at Ruhr University Bochum (EKS24072017).

### Experimental Setup

**Figure 1:**
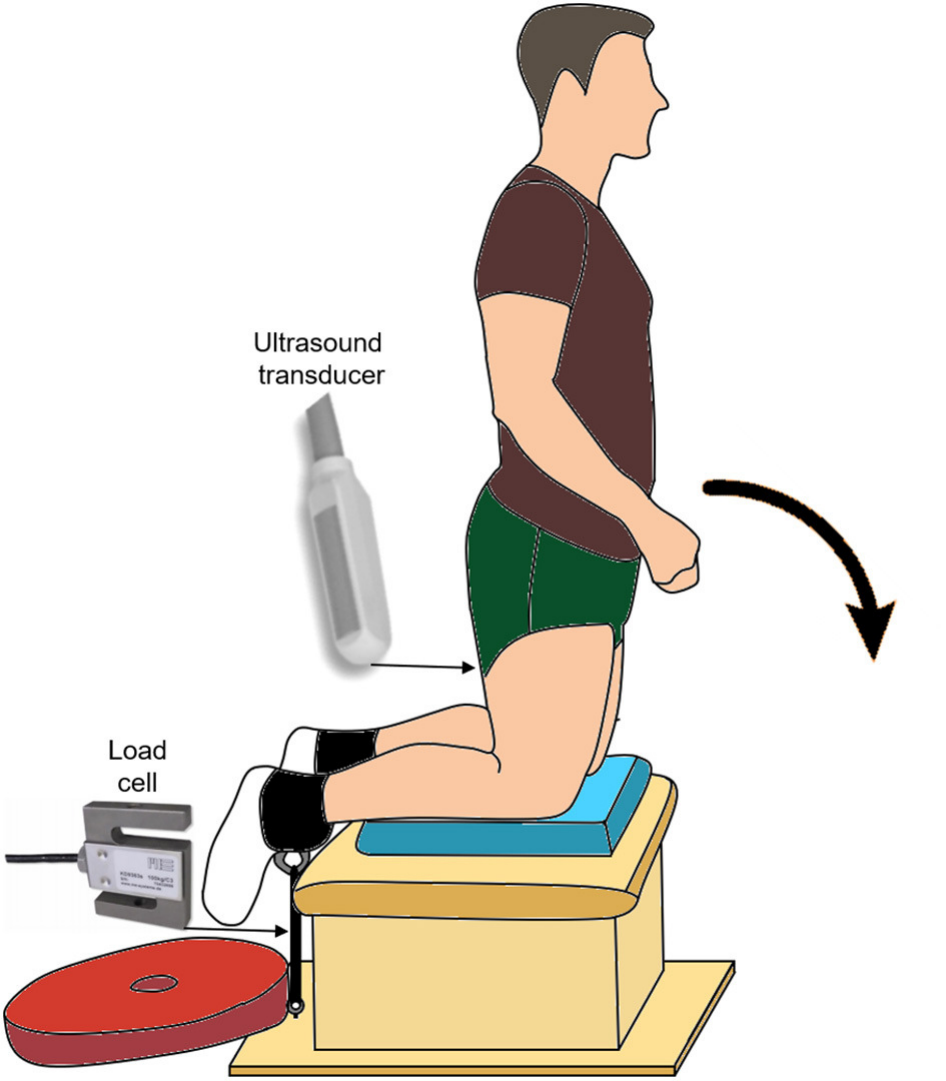
Experimental setup showing the beginning posture for the Nordic hamstring exercise (NHE). The participants' end posture was slightly below horizontal, as their upper body was supported on a box (not shown here) that was half the height of the box under their shanks. The approximate location and type of ultrasound transducer, as well as the approximate location and type of load cell, are also shown.

The custom-made experimental setup can be seen in Figure 1. Briefly, participants performed the NHE and prone maximal voluntary contraction trials in this setup with their shanks positioned on a padded box and parallel to the ground. As the participants' feet hung off the box, individual straps (MA1026; Fitness Express 24, GER) could be secured around their ankles. The center of each strap was first aligned with the lateral malleolus of each leg, and then the straps were suspended vertically and connected distally to an adjustable strap via a carabiner. This adjustable strap was distally connected to a horizontal wooden plate that acted as an immovable resistance for participants to bilaterally pull against while they activated their hamstring muscles during the NHE.

### Experimental Tasks

Participants started the NHE in a tall kneel position at around 90° knee flexion and 0° hip flexion (Figure 1). Participants were instructed to lower themselves into the prone position (i.e. 0° knee and hip flexion) as slowly as possible, while keeping their hips and trunk in neutral positions and their arms across their chest. The NHE ended when the participants used their hands to lower their chest onto a box that was half the height as the box under their shanks.

### Experimental Protocol

Participants performed five consecutive prone submaximal knee flexion contractions (1-s duration, 1-s rest) with 80% of their maximal effort to precondition the hamstring MTUs (Maganaris et al., 2002). The warmup then consisted of at least two maximal-effort prone voluntary contractions (3-s duration, 60-s rest) and three sets of two NHE trials with submaximal efforts at 50, 70, and 90% of the participants' perceived maximum. At least two sets of three maximal-effort NHE trials (120-s rest between sets) followed the warmup, and up to three additional trials were performed if the highest peak NHE forces from two trials were not within 5% of each other.

### Measurements

#### External Reaction Force

External reaction force was measured using a S-type load cell (KD9363s; ME-Messsysteme GmbH, GER) that was in series between the left ankle and ground within a setup (Figure 1) that allowed the long axis of the load cell to change with the direction of the applied force. The force signal was amplified using a custom-built amplifier and calibrated prior to testing. Force was sampled at 1,000 Hz using a 16-bit analog-to-digital converter (Power1401; Cambridge Electronic Design, UK) and paired data acquisition system (Spike2). This system was used to collect and synchronize all other data.

#### Knee Joint Angle

Knee flexion angle was measured at 1,000 Hz using a custom-made digital goniometer that consisted of two rotatable plastic arms connected to a potentiometer (Vishay, USA). The goniometer was calibrated prior to testing, and it had a precision of 1.5°. The arms of the goniometer were aligned with the longitudinal axes of the femur and fibula and fixed on the lateral aspect of the left lower limb with surgical tape (3M Transpore, GER) once the potentiometer was aligned with the axis of rotation of the knee as approximated by the femur's lateral condyle.

#### Muscle Activity

Muscle activities of the left-sided BF and semitendinosus muscles were recorded with surface electromyography at 2,000 Hz using bipolar electrodes (Ag/AgCl, H124SG; Covidien Kendall, GER) placed 2 cm apart over the distal BF muscle belly and most prominent bulge of the semitendinosus muscle belly, respectively. The distal location of BF electrodes (which were still located over the long head and not short head of the muscle as confirmed via ultrasound imaging) was due to the immediate proximal location of the ultrasound transducer. A single reference electrode was placed over the knee's lateral joint line, and electrode placement was preceded by standard skin preparation. Electromyography (EMG) signals were band-pass analog filtered between 10 and 500 Hz and amplified 500 to 2,000 times depending on signal strength via the AnEMG12 amplifier (OT Bioelettronica, IT).

#### Muscle Fascicle Behavior

Muscle architecture of the left-sided BF was imaged at 61.5 Hz with two-dimensional B-mode ultrasound using a 60-mm linear, flat, 128-element transducer (LV7.5/60/128Z-2, 6 MHz, 50/65-mm image depth; Telemed, Lithuania) connected to a PC-based ultrasound system running paired Echo Wave II software. The ultrasound transducer was placed in a custom-made cutout to minimize local hamstring muscle compression and initially oriented in the transverse plane on the posterior aspect of the thigh at the midpoint between the ischial tuberosity and lateral condyle, which were located via palpation. BF muscle borders were confirmed by having participants perform active knee and hip flexions and extensions. The transducer was subsequently rotated 90° about its yaw axis to image BF muscle fascicles and then translated and rotated about its pitch and roll axes until the clearest images of continuous muscle fascicles and parallel aponeuroses could be obtained from BF's superficial compartment during a knee flexion contraction. The transducer was fixed to the posterior thigh using self-adhesive bandage (Medichill, UK), which restricted transducer movement relative to the skin.

Muscle fascicle length of one representative fascicle from BF's superficial compartment was calculated offline in Matlab (R2019b; MathWorks, USA) using the previously described tracking software and procedures of UltraTrack (Farris and Lichtwark, 2016) and a recently developed custom software tool (Drazan et al., 2019). The former approach calculates a set of optical flow vectors and uses a least-squares solution to average the optical flow approximations from within a user-defined region of interest to detect sub-millimeter fascicle length changes (Day et al., 2017). UltraTrack provides a reliable semi-automated alternative to manually determining fascicle lengths in each ultrasound image (Gillett et al., 2013). The latter approach uses the same optical flow algorithm, but only tracks features that are suitable for the tracking algorithm to directly track features of the user-defined muscle fascicle and aponeuroses and will be referred to as PointTrack from here on. PointTrack has been reported to exhibit lower root-mean-square errors between automatically determined and manually defined fascicle lengths compared with UltraTrack (Drazan et al., 2019). Fascicle length changes were determined using both tracking algorithms to improve the robustness of the fascicle length change results.

To determine MTU length changes during the NHE, normalized MTU lengths were first calculated by using the regression equation and corresponding BF coefficients reported in Hawkins and Hull (1990). Absolute MTU lengths were then calculated by multiplying normalized MTU lengths by the thigh length of the respective participant. As hip angles were not measured, the hip coefficient in the regression equation (Hawkins and Hull, 1990) was set to zero.

### Data Analysis

All data were processed offline using custom-written scripts in Matlab. External reaction forces were filtered using a second-order 20 Hz low-pass Butterworth filter corrected for two passes (Winter, 2009). Knee flexion angles and the x–y coordinates of tracked fascicle endpoints were filtered as above, but with a 6 Hz cutoff frequency. Fascicle lengths were recalculated from the filtered x–y coordinates to limit the influence of aponeurosis orientation changes on the fascicle length measurements, and fascicle velocity (positive = lengthening) was calculated by differentiating the filtered fascicle length signal with respect to 2*t* (where *t* = time) (Winter, 2009). Knee angular velocity (positive = knee extension) was also calculated by differentiating the filtered knee joint angle signal with respect to 2*t* (Winter, 2009). EMG signals had the DC offset removed and were smoothed with a zero-phase 0.05-s root-mean-square amplitude calculation.

The NHE trial with the highest peak force from a minimum of six to a maximum of nine trials across participants was included in the analysis of kinematic, kinetic, and muscle activity data. Muscle activity and force data were normalized to the peak values from that respective trial for analysis. Fascicle and MTU length changes and velocities were calculated from the same trials after all signals were down sampled to the ultrasound sampling rate (61.5 Hz). Fascicle and MTU data were excluded from statistical analysis if the magnitude of muscle lengthening along the muscle's line of action (i.e. fascicle length multiplied by the cosine of the angle between the fascicle and its intermediate aponeurosis) exceeded the magnitude of MTU lengthening by more than 9 mm. This value was based on the maximum fascicle length measurement error we expected with the ultrasound transducer perpendicular to the local tangent plane to the skin (i.e. 0° transducer tilt) and a changing misalignment between image and fascicle planes of up to 22° (Bolsterlee et al., 2016) during the period when fascicle lengths were tracked. This 9-mm estimate was relatively conservative because 22° of misalignment is double what has been previously found for real ultrasound images of the human medial gastrocnemius (Bolsterlee et al., 2015); however, we decided to be conservative because we imaged a muscle with a more complicated architecture and because there are currently no data showing how much misalignment between image and fascicle planes changes during dynamic movements. If a 9-mm positive difference between fascicle and MTU lengthening magnitudes was observed, it was deemed that unmeasured hip flexion contributed to additional unmeasured lengthening of the MTU, which was an exclusion criterion because it indicated that the participants did not adequately control their hip angle during the NHE. Fascicle and MTU data were also excluded from analysis if the direction of net fascicle length change (i.e. lengthening or shortening) determined by the two ultrasound tracking algorithms was not the same since this limited the robustness of the fascicle length change results.

### Statistics

Statistical analyses were performed using Prism 9 software (GraphPad, USA). A parametric test was performed after the normality of data for one-sample *t*-tests or the normality of residuals for within-subject comparisons was not rejected at an alpha level of 5% according to Shapiro–Wilk tests (or D'Agostino and Pearson tests in datasets with tied observations). One-sample *t*-tests were used to compare the net fascicle length changes detected by the two earlier-described tracking algorithms to a constant of zero, and the family-wise alpha level was controlled at 5% with a Bonferroni correction. One-sample *t*-tests were also used to compare the mean fascicle velocities determined by the two tracking algorithms to a constant of zero, and the family-wise alpha level was again controlled at 5% with a Bonferroni correction. Paired *t*-tests were used to assess if the differences in net fascicle length change and mean fascicle velocity determined by the two tracking algorithms were surprising assuming no differences. Cohen's *d*_*z*_ (one-sample *t*-test) and Hedges's *g*_*av*_ (paired *t*-test) were calculated according to the formulas in Lakens (2013), and confidence intervals (CIs) were computed on *d* using the non-central *t* method (Kelley, 2007). Data are reported as mean ± standard deviation in the text.

## Results

### Data Exclusion

Fascicle and MTU data from two participants (P7 and P13) were excluded from analysis because muscle lengthening along the muscle's line of action exceeded MTU lengthening by more than 9 mm, indicating uncontrolled hip flexion. Fascicle and MTU data from an additional two participants (P3 and P9) were excluded from analysis because the direction of net fascicle length change determined by the two tracking algorithms was different. Knee flexion angle data from one participant (P13) was excluded due to implausible values (e.g., a 180° knee flexion angle) during the NHE, and semitendinosus muscle activity data from one participant (P4) was excluded due to signal clipping.

### Kinematics and Kinetics

**Figure 2:**
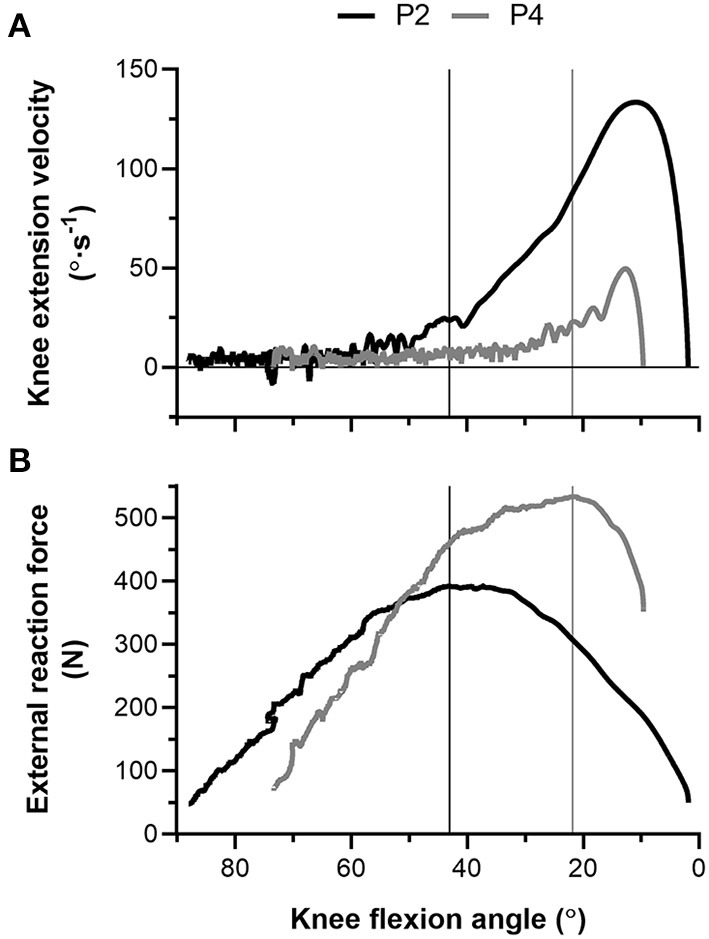
**(A)** Left-sided knee extension velocity and **(B)** external reaction force-knee flexion angle traces during the Nordic hamstring exercise (NHE) trial with the highest peak force from two participants (P2, black; P4, gray). The vertical black (P2) and gray (P4) lines show the difference in knee flexion angle when peak force was attained between the two participants. Note the fluctuation in knee extension velocity around zero at more flexed knee angles during the NHE, and note the higher forces and lower knee extension velocities at more extended knee angles in the stronger participant (P4).

**Figure 3:**
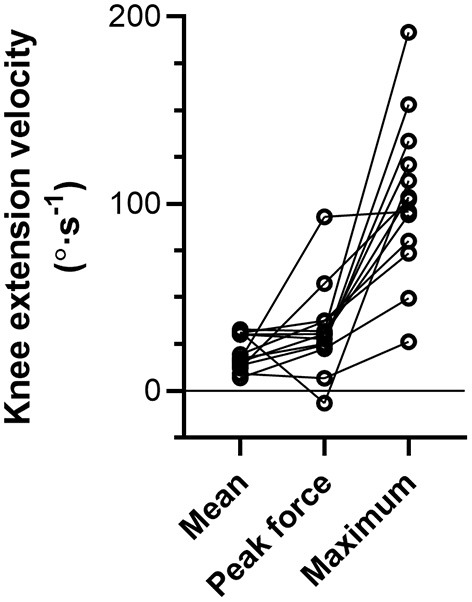
Individual left-sided knee extension velocities across participants (*n* = 13) during the Nordic hamstring exercise (NHE) trial with the highest peak force. Mean refers to the mean velocity over the period when knee extension velocity was positive, peak force refers to the instantaneous velocity at peak NHE force, and maximum refers to the peak knee extension velocity during the NHE. Solid lines link the same participant, and descriptive statistics are reported in the Results section.

**Figure 4:**
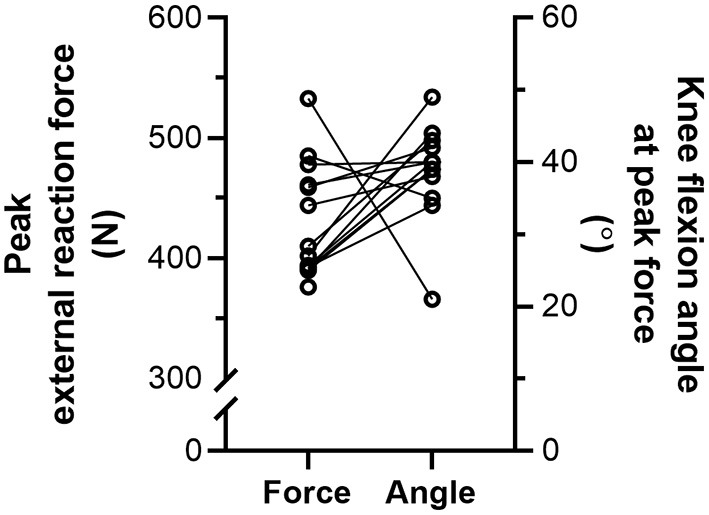
Individual left-sided peak external reaction forces (*N* = 14) and knee flexion angles (*n* = 13) at peak force during the Nordic hamstring exercise (NHE) trial with the highest peak force. Solid lines link the same participant, and descriptive statistics are reported in the Results section.

During the NHE (Figure 1), knee extension velocity first became and remained positive (Figure 2) from a knee flexion angle of 62 ± 15° up until a knee flexion angle of 6 ± 11° (*n* = 13). The mean knee extension velocity over this period was 20.5 ± 9.3°·s^−1^ (Figure 3). The duration knee extension velocity remained positive at 3.4 ± 2.3 s (range: 0.9–7.9 s), and external reaction force reached a maximum of 429 ± 47 N (*n* = 14, Figure 4) during this period in all participants except one (Figure 3). At peak NHE force, the mean knee flexion angle was 38 ± 7° (Figure 4), and the mean knee extension velocity was 32.2 ± 23.7°·s^−1^ (Figure 3). At 0.5 ± 0.4 s after peak force, knee extension velocity reached a maximum of 102.8 ± 42.8°·s^−1^ (Figure 3) at a knee flexion angle of 17 ± 10° (range: 3–37°).

### Muscle Fascicle Behavior

**Figure 5:**
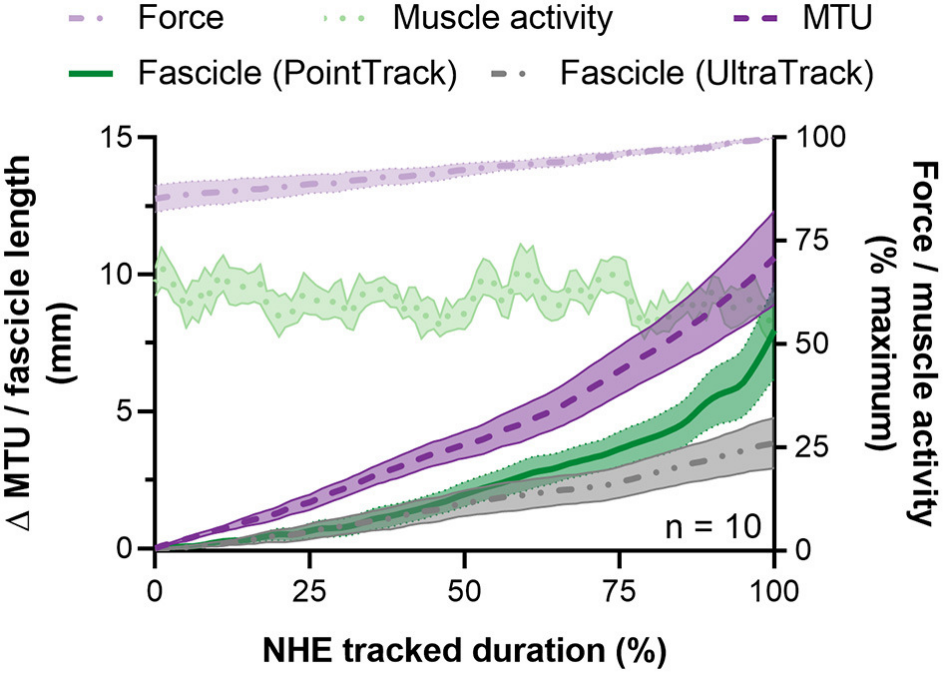
Mean ± SEM left-sided biceps femoris long head (BF) fascicle length and muscle-tendon unit (MTU) length changes (left y-axis) and normalized external reaction forces and BF EMG root-mean-square amplitudes (right y-axis) during the Nordic hamstring exercise (NHE) from 85 to 100% of peak force. Due to the different durations that fascicle lengths were tracked up until peak force, all data from one NHE trial across participants were time normalized to 100 data points and averaged. Fascicle length changes determined from the two tracking algorithms described in the Materials and Methods section are shown.

**Figure 6:**
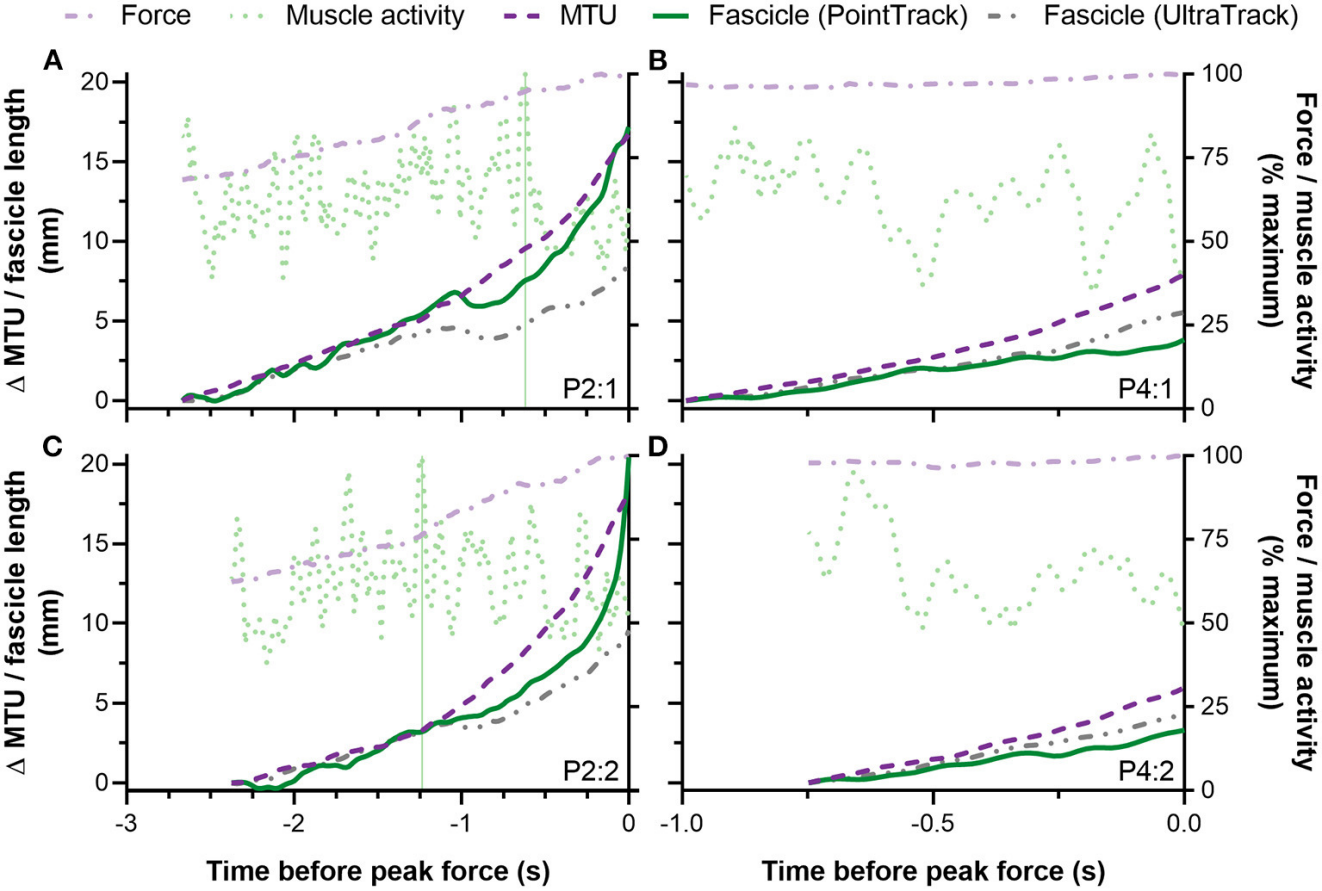
Individual traces from two participants **(A–D)** showing left-sided biceps femoris long head (BF) fascicle length and muscle-tendon unit (MTU) length changes (left y-axis) and normalized external reaction forces and BF EMG root-mean-square amplitudes (right y-axis) during the Nordic hamstring exercise (NHE) up until peak force. The top panels **(A,B)** show data from the NHE trial with the highest peak force, and the bottom panels **(C,D)** show data from the NHE trial with a similar, but lower peak force (<5% difference). Only the data from the top row were included in the statistical analysis. Note the similar patterns of fascicle and MTU length change and rates of force development between trials from the same participant. Also note the similar fascicle length changes determined from the same tracking algorithm between trials from the same participant. The vertical green lines in **(A,C)** show the difference in maximum BF muscle activity timings relative to peak force between two trials from the same participant. The other participant **(B,D)** did not attain their maximum BF muscle activity over the period when fascicle lengths were tracked in either trial.

**Figure 7:**
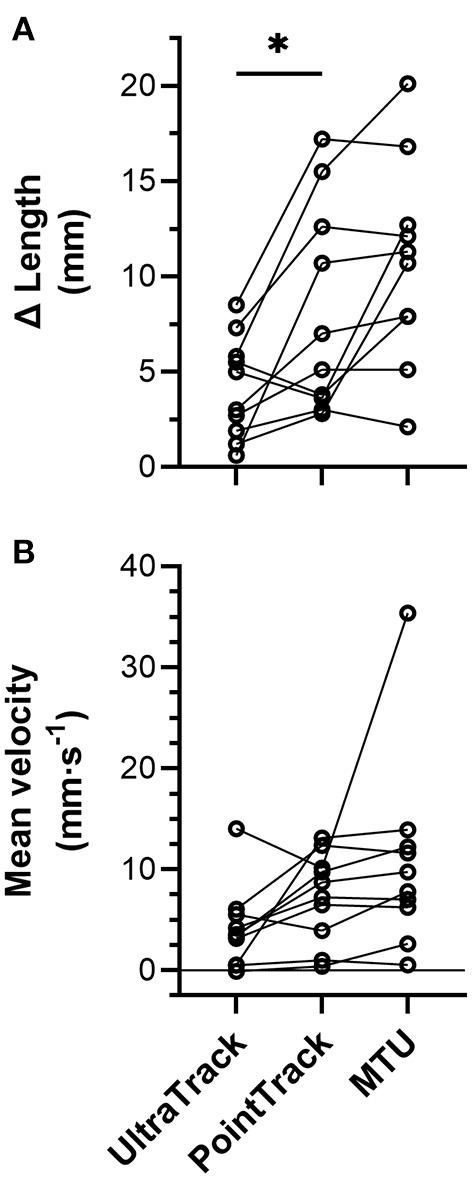
Individual (*n* = 10) **(A)** left-sided biceps femoris long head (BF) muscle fascicle length and muscle-tendon unit (MTU) length change amplitudes and **(B)** mean velocities over the period when fascicle lengths were tracked from ~85 to 100% of peak force during the Nordic hamstring exercise (NHE). Solid lines link the same participant, and descriptive statistics are reported in the *Results* section. *Indicates a significant difference between conditions (*p* < 0.05).

From the NHE starting position, BF muscle fascicles initially shortened, but the amount of shortening was not quantified due to substantial out-of-plane motion of muscle fascicles with respect to the ultrasound imaging plane. Fascicle length changes (*n* = 10) were quantified during the NHE from 85 ± 10% of peak force (range: 67–97%) until peak force (443 ± 49 N, range: 390–533 N; Figure 5), which corresponded to knee flexion angles from 53 ± 11° to 37 ± 6° and a duration of 1.6 ± 1.2 s (range: 0.3–4.0 s or 22–250 ultrasound images). Over this period, knee extension velocity was 16.6 ± 14.8°·s^−1^ (range: 0.7–54.5°·s^−1^), and MTU and fascicle length change patterns were subjectively similar between two NHE trials with similar peak forces (<5% difference) within the same participant (Figure 6; note that only data from the trial with the highest peak force was used in the analysis). The BF MTU lengthened by 10.7 ± 5.3 mm (Figure 7A), and both ultrasound tracking algorithms detected net fascicle lengthening. The fascicle lengthening detected by Over the same period as above, the BF MTU velocity was 10.7 ± 9.6 mm·s^−1^ (Figure 7B), and the mean fascicle velocity determined by both tracking algorithms was positive. The mean fascicle velocity detected by UltraTrack was significantly different from zero (*t*_(9)_ = 3.16, *p* = 0.023, 4.1 ± 4.1 mm·s^−1^ [95% CI: 1.2 to 7.0 mm·s^−1^], *d*_*z*_ = 1.00 [0.21 to 1.75]), as was the mean fascicle velocity detected by PointTrack (*t*_(9)_ = 5.24, *p* = 0.001, 7.3 ± 4.4 mm·s^−1^ [95% CI: 4.2 to 10.5 mm·s^−1^], *d*_*z*_ = 1.66 [0.66 to 2.62]). These can be considered large effects. There was no significant difference between tracking algorithms [*t*_(9)_ = 2.17, *p* = 0.058, 3.2 ± 4.7 mm·s^−1^ [95% CI: −0.1 to 6.6 mm·s^−1^], *g*_*av*_ = 0.70 [−0.03 to 1.51], *r* = 0.39; Figure 7B], but this can be considered a medium effect.

UltraTrack was significantly different from zero (*t*_(9)_ = 4.93, *p* = 0.002, 4.2 ± 2.7 mm [95% CI: 2.2 to 6.1 mm], *d*_*z*_ = 1.56 [0.60 to 2.48]), as was the fascicle lengthening detected by PointTrack (*t*_(9)_ = 4.72, *p* = 0.002, 8.1 ± 5.5 mm [95% CI: 4.2 to 12.0 mm], *d*_*z*_ = 1.49 [0.55 to 2.39]). These can be considered large effects. There was a significant difference between tracking algorithms [*t*_(9)_ = 2.88, *p* = 0.018, 4.0 ± 4.4 mm [95% CI: 0.9 to 7.1 mm], *g*_*av*_ = 0.85 [0.15 to 1.67], *r* = 0.61; Figure 7A], and this can be considered a large effect.

### Muscle Activity

**Figure 8:**
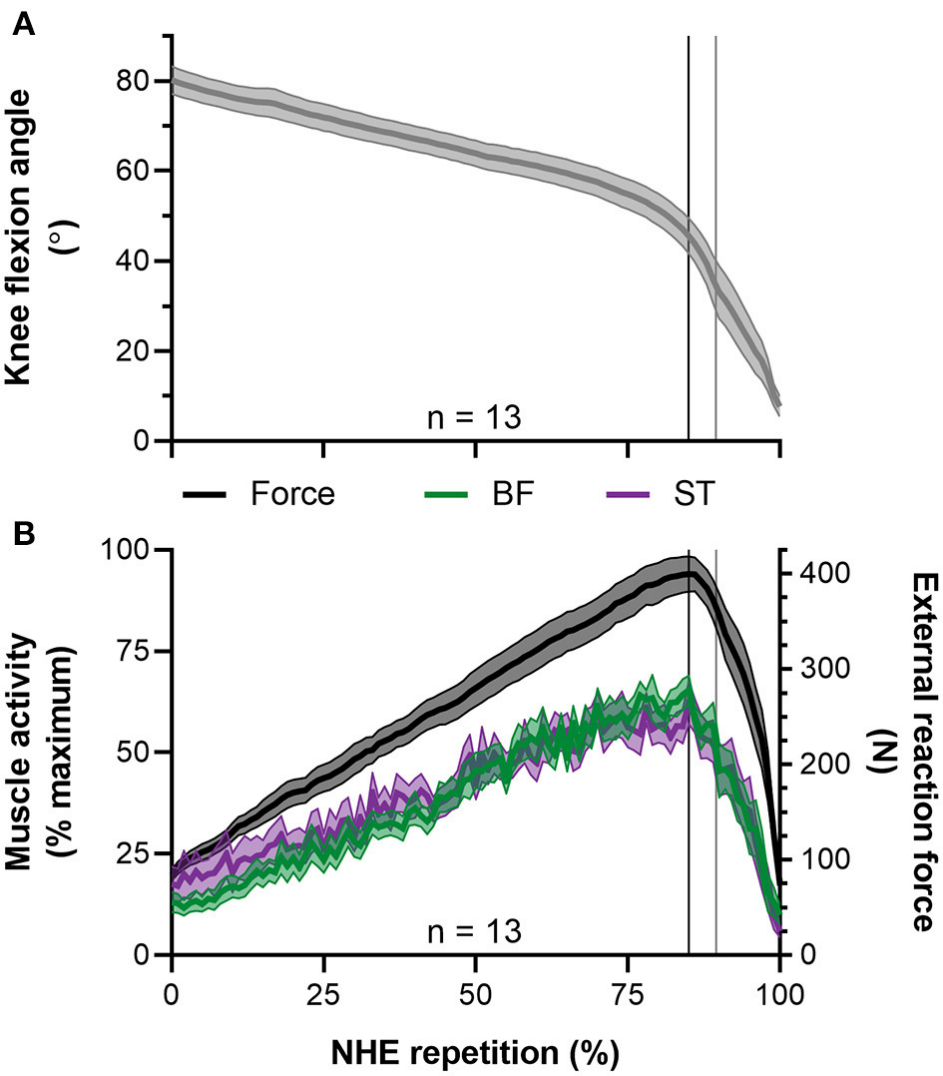
Mean ± SEM left-sided **(A)** knee flexion angles and **(B)** normalized biceps femoris (BF) and semitendinosus (ST) EMG root-mean-square amplitudes (left y-axis) and external reaction forces (right y-axis) during the Nordic hamstring exercise (NHE). Due to the different durations that participants performed the NHE, all data from the NHE trial with the highest peak force across participants were clipped between 10% of peak NHE force before and after peak force, time normalized to 100 data points, and then averaged. The vertical black lines indicate when peak NHE force and peak muscle activities occurred. The vertical gray lines indicate when peak knee extension velocity occurred.

During the period when fascicle lengths were tracked, the minimum, mean, and maximum BF root-mean-square amplitudes normalized to the maximum amplitude attained in the same NHE trial were 34 ± 3% (range: 29–42%), 59 ± 6% (range: 47–65%), and 90 ± 11% of maximum (range: 70–100%, *n* = 10), respectively. These moderate-to-high BF muscle activity levels, in combination with the BF muscle activity traces shown in Figures 5 and 8 indicate it is unlikely that a reduction in muscle activity level largely contributed to the BF fascicle lengthening detected by the two tracking algorithms up until peak NHE force. The BF muscle activity pattern was similar to the semitendinosus muscle activity pattern (Figure 8) over the same period, which had minimum, mean, and maximum normalized root-mean-square amplitudes of 31 ± 5% (range: 24–39%), 56 ± 6% (range: 48–62%), and 88 ± 13% of maximum (range: 60–100%, *n* = 9), respectively.

## Discussion

The aim of this study was to determine whether eccentric hamstring muscle action occurs during the NHE in Nordic novices. For this purpose, BF muscle fascicle lengths were imaged with B-mode ultrasound, and fascicle length changes were quantified using two different ultrasound tracking algorithms. Due to difficulties in quantifying BF muscle fascicle length changes during the start of the NHE, fascicle length changes were only quantified from on average 85% of peak NHE force until peak NHE force, which corresponded to knee flexion angles from 53 to 37° and a mean duration of 1.6 s. Over this period, BF fascicles significantly lengthened and had a mean positive velocity while mean BF muscle activity was 59% of its maximum during the NHE. These findings provide the first direct experimental support that the NHE causes eccentric action of at least one hamstring muscle when performed by Nordic novices, which might contribute to the increase in resting BF muscle fascicle length (Bourne et al., 2017; Alonso-Fernandez et al., 2018; Presland et al., 2018; Pollard et al., 2019) and reduction in running-related injury risk (Al Attar et al., 2017; van Dyk et al., 2019) that have previously been reported following NHE training.

### Kinematics and Kinetics

The NHE knee kinematics observed in this study are similar to previous reports in terms of the knee flexion angle at peak force (Delahunt et al., 2016), the mean knee extension velocity (Alt et al., 2018), and the peak knee extension velocity (Ditroilo et al., 2013; Delahunt et al., 2016). Although the participant group was previously unfamiliar with performing the NHE, the level of NHE strength (429 ± 47 N) was higher compared with that of elite Australian footballers (301 ± 84 N) (Opar et al., 2015), professional soccer players (310 ± 73 N) (Timmins et al., 2016), and elite (367 ± 77 N) and sub-elite (388 ± 96 N) Rugby Union players (Bourne et al., 2015). Initially, it was expected that this might be because the participants in this study were more familiarized with the NHE as they performed six to nine maximal-effort trials and most frequently attained their highest peak NHE force in the sixth trial, whereas the previous studies had athletes perform only three maximal-effort repetitions. However, the mean peak force from the participants' first three trials in this study was 406 ± 55 N and there thus may have been a potential selection bias from this study's relatively small sample size (*N* = 14 vs. *N* = 131–210). Another possibility is that the higher peak forces measured in this study could be due to differences in force transducer placement and the rigidity of the experimental setup compared with previous studies.

### Muscle Fascicle Behavior

The relatively high strength level of the participants, in combination with the mean 59% of maximum BF muscle activity level, the 17°·s^−1^ mean knee extension velocity, and the 11 mm·s^−1^ mean positive BF MTU velocity determined over the period when fascicle length changes were tracked, likely contributed to the net BF muscle fascicle lengthening detected. For example, it is unlikely that an eccentric BF muscle action would have been observed if any participant attained peak NHE force when BF MTU velocity was zero (i.e. during a brief isometric-hold phase in the NHE when a given posture could be maintained). This is because muscle fascicle velocity cannot be positive when MTU velocity is zero and muscle activity is constant. Only one participant in this study showed a mean MTU velocity close to zero (0.5 mm·s^−1^), which coincided with 2 mm of MTU lengthening and 2–3 mm of BF muscle fascicle lengthening. Because fascicle lengthening just exceeded MTU lengthening (as estimated from knee flexion angles alone), and slightly higher positive fascicle velocities (0.5–1.0 mm·s^−1^) were observed, it is likely that this participant exhibited hip flexion over the period when fascicle lengths were tracked, which could be examined in future studies with motion capture. It is therefore not possible to know if the eccentric BF muscle action was due to hip flexion, knee extension, or a combination of the two in this study.

Despite an eccentric BF muscle action being observed from the analyzed fascicle data (*n* = 10) from ~85 to 100% of peak NHE force, this does not mean that the BF muscle always acted eccentrically during this phase. For example, for brief periods, BF muscle fascicle shortening was observed during MTU lengthening (e.g., Figure 6A at −1 s) or muscle fascicles remained isometric (e.g., Figure 6C at −1.75 s). This decoupling between muscle fascicle and MTU length changes was expected due to MTU compliance, but the decoupling was not clearly pronounced like it is during the mid-stance phase of overground walking (Cronin et al., 2013). This is likely to be because of the much higher muscle activities attained during maximal-effort NHE trials and the subsequent reduction in MTU compliance that occurs at high forces (Scott and Loeb, 1995). At lower forces during the NHE (e.g., 0–50% of maximum), when the MTU is more compliant, it is thus speculated that muscle fascicle shortening would occur despite MTU lengthening, which is generally what was observed in the ultrasound images but not able to be quantified. It is also speculated that the muscle adaptations that arise from NHE training are likely to vary between individuals because of different between-subject, yet relatively consistent within-subject, muscle fascicle behaviors (Figure 6). Ideally, these between-subject differences in muscle fascicle and MTU behavior should be quantified and considered when relationships between a strength exercise and region-specific training-induced muscle adaptations are investigated.

#### Automated Muscle Fascicle Tracking

Two ultrasound tracking algorithms were used to determine muscle fascicle length changes during the NHE to ensure that the direction of net length length change observed was not erroneous. In two participants, the direction of net fascicle length change was different between the two tracking algorithms [2–3 mm of shortening (UltraTrack) vs. 5–6 mm of lengthening (PointTrack)] and so the data were excluded from analysis, despite visual inspection suggesting net fascicle lengthening. The finding that BF fascicle lengthening magnitudes determined by UltraTrack (Farris and Lichtwark, 2016) were significantly less than those determined by PointTrack (Drazan et al., 2019) indicates that UltraTrack might underestimate fascicle length changes when there are large frame-to-frame fascicle displacements. While this issue could have been mitigated or avoided by using a faster ultrasound frame rate (>62 Hz), this was not possible in the current study due to hardware limitations. As PointTrack subjectively followed frame-to-frame fascicle displacements more closely (Supplementary Video 1), the fascicle lengthening magnitudes are likely to be more accurate than those estimated by UltraTrack. This is because image pyramids can be used with PointTrack to reduce the resolution of images to track larger pixel motions outside of the pixel neighborhood.

### Limitations

All measurements were made from the left leg in this study, but due to the bilateral nature of the NHE, we believe that similar results would be obtained from the right leg in our participant group. Knee joint torques were not estimated because our relatively limited experimental setup resulted in many assumptions being required for an inverse dynamics analysis. Only males were tested as the prospective evidence supporting the effectiveness of NHE training for reducing hamstring injury risk comes from this group (Arnason et al., 2008; Petersen et al., 2011; Seagrave et al., 2014; van der Horst et al., 2015; Timmins et al., 2016). BF muscle fascicle length changes could only be quantified two-dimensionally from a limited muscle region in a subset of participants (10 from 14) due to difficulties with aligning the ultrasound imaging plane with the muscle fascicle plane throughout the NHE. Fascicle strains were not estimated and absolute fascicle lengths are not reported due to uncertainty in the accuracy of absolute fascicle length measurements (Franchi et al., 2020). Relationships between peak NHE force and BF muscle fascicle behavior, as well as between NHE kinematics and muscle fascicle behavior, were also not quantified due to the low sample size. The magnitude of BF muscle fascicle lengthening detected was likely to be influenced by the participants' NHE familiarization within the testing session, which may not have been sufficient in one individual with analyzed fascicle data, considering that none of the six NHE trials had similar peak forces (<10% difference) relative to the maximum force from the seventh trial. However, because all other participants showed at least two NHE trials with similar maximum forces (<5% difference), it is unlikely that inadequate task familiarization affected the study's main conclusion of eccentric BF muscle action in Nordic novices.

## Conclusions

In conclusion, two different ultrasound tracking algorithms detected net BF muscle fascicle lengthening and a positive mean fascicle velocity from ~85 to 100% of peak force during the NHE. This fascicle behavior was observed while knee extension velocity was positive over knee flexion angles from 53 to 37°. Despite some individual cases of brief isometric fascicle behavior and brief fascicle shortening during BF MTU lengthening, the relatively high mean muscle activity level observed (59% of maximum) during net fascicle lengthening likely positive MTU velocity, likely prevented pronounced decoupling between muscle fascicle and MTU length changes as MTU compliance is reduced at high forces (Scott and Loeb, 1995). These results support the notion that the NHE causes a predominantly eccentric muscle action of one of the hamstrings under high forces in Nordic novices. This eccentric muscle action might contribute to the increase in resting BF muscle fascicle length (Bourne et al., 2017; Alonso-Fernandez et al., 2018; Presland et al., 2018; Pollard et al., 2019) and reduction in running-related injury risk (Al Attar et al., 2017; van Dyk et al., 2019) that have previously been reported following NHE training, but this deserves further study. Additionally, whether an eccentric BF muscle action occurs in individuals accustomed to the NHE remains to be tested.

## Data Availability Statement

The raw data supporting the conclusions of this article will be made available by the authors, without undue reservation.

## Ethics Statement

This study involving human participants was reviewed and approved by the Faculty of Sport Science Ethics Committee at Ruhr University Bochum (EKS24072017). The participants provided their written informed consent to participate in this study.

## Author Contributions

BR contributed to conception and design of the study, acquisition of data, data analysis, and interpretation and drafted the manuscript. RB contributed to conception and design of the study, collected the data, contributed to data analysis and interpretation, and revised the manuscript critically. DH contributed to conception and design of the study and data interpretation and revised the manuscript critically. All authors gave the final approval for publication.

## Conflict of Interest

The authors declare that the research was conducted in the absence of any commercial or financial relationships that could be construed as a potential conflict of interest.

## Abbreviations

BF: biceps femoris long head
MTU: muscle-tendon unit
NHE: Nordic hamstring exercise.
